# Implementing evidence-based practices to improve primary care for high-risk patients: study protocol for the VA high-RIsk VETerans (RIVET) type III effectiveness-implementation trial

**DOI:** 10.1186/s43058-024-00613-9

**Published:** 2024-07-15

**Authors:** Elvira E. Jimenez, Ann-Marie Rosland, Susan E. Stockdale, Ashok Reddy, Michelle S. Wong, Natasha Torrence, Alexis Huynh, Evelyn T. Chang

**Affiliations:** 1 Center for the Study of Healthcare Innovation, Implementation & Policy (CSHIIP), VA Greater Los Angeles Healthcare System, 11301 Wilshire Blvd, Los Angeles, CA 90073 USA; 2 Department of Neurology, David Gefen School of Medicine, University of California Los Angeles (UCLA), 760 Westwood Plaza, Los Angeles, CA 90095 USA; 3 Center for Health Equity Research and Promotion (CHERP), VA Pittsburgh Healthcare System, 1 University Dr, Pittsburgh, PA 15240 USA; 4 Caring for Complex Chronic Conditions Research Center & Division of General Internal Medicine, School of Medicine, University of Pittsburgh, 3550 Terrace St, Pittsburgh, PA 15213 USA; 5 Department of Medicine, Division of General Internal Medicine, Harborview Medical Center, University of Washington, 325 Ninth Ave, Box 359780, Seattle, WA 98104 USA; 6 Center for Veteran–Centered and Value–Driven Care, VA Puget Sound Health Care System, 1660 S Columbian Way, Seattle, WA 98108 USA; 7 Division of General Internal Medicine, Department of Medicine, David Gefen School of Medicine, UCLA, 740 Charles E Young Dr S, Los Angeles, CA 90095 USA; 8 Department of Psychiatry and Biobehavioral Sciences, David Geffen School of Medicine, University of California Los Angeles (UCLA), 760 Westwood Plaza, Los Angeles, CA 90095 USA

**Keywords:** Primary Health Care, Multimorbidity, Medication Adherence, Needs Assessment

## Abstract

**Background:**

Patients with significant multimorbidity and other factors that make healthcare challenging to access and coordinate are at high risk for poor health outcomes. Although most (93%) of Veterans’ Health Administration (VHA) patients at high risk for hospitalization or death (“high-risk Veterans”) are primarily managed by primary care teams, few of these teams have implemented evidence-based practices (EBPs) known to improve outcomes for the high-risk patient population’s complex healthcare issues. Effective implementation strategies could increase adoption of these EBPs in primary care; however, the most effective implementation strategies to increase evidence-based care for high-risk patients are unknown.

The high-RIsk VETerans (RIVET) Quality Enhancement Research Initiative (QUERI) will compare two variants of Evidence-Based Quality Improvement (EBQI) strategies to implement two distinct EBPs for high-risk Veterans: individual coaching (EBQI-IC; tailored training with individual implementation sites to meet site-specific needs) versus learning collaborative (EBQI-LC; implementation sites trained in groups to encourage collaboration among sites). One EBP, Comprehensive Assessment and Care Planning (CACP), guides teams in addressing patients’ cognitive, functional, and social needs through a comprehensive care plan. The other EBP, Medication Adherence Assessment (MAA), addresses common challenges to medication adherence using a patient-centered approach.

**Methods:**

We will recruit and randomize 16 sites to either EBQI-IC or EBQI-LC to implement one of the EBPs, chosen by the site. Each site will have a site champion (front-line staff) who will participate in 18 months of EBQI facilitation.

**Analysis:**

We will use a mixed-methods type 3 hybrid Effectiveness-Implementation trial to test EBQI-IC versus EBQI-LC versus usual care using a Concurrent Stepped Wedge design. We will use the Practical, Robust Implementation and Sustainability Model (PRISM) framework to compare and evaluate Reach, Effectiveness, Adoption, Implementation, and costs. We will then assess the maintenance/sustainment and spread of both EBPs in primary care after the 18-month implementation period. Our primary outcome will be Reach, measured by the percentage of eligible high-risk patients who received the EBP.

**Discussion:**

Our study will identify which implementation strategy is most effective overall, and under various contexts, accounting for unique barriers, facilitators, EBP characteristics, and adaptations. Ultimately this study will identify ways for primary care clinics and teams to choose implementation strategies that can improve care and outcomes for patients with complex healthcare needs.

**Trial registration:**

ClinicalTrials.gov, NCT05050643. Registered September 9th, 2021, https://clinicaltrials.gov/study/NCT05050643

**Protocol version:**

This protocol is Version 1.0 which was created on 6/3/2020.

Contributions to the literature

- The first study to compare the effectiveness of Evidence-Based Quality Improvement (EBQI) conducted individually with one site or conducted with a group of sites.
- Implementation of evidence-based practices to improve care for high-risk patients in primary care.

## Background

Patients who are at the highest risk for hospitalization (“high-risk patients”) are a heterogenous subset of patients who have significant multimorbidity and pose the most significant medical challenges within any healthcare organization [1]. These patients are at high risk for poor health outcomes and account for the majority of the Veterans Health Administration (VHA) healthcare costs [2], similar to other healthcare systems [3, 4]. Previous Medicare demonstrations, such as advanced primary care home models called Comprehensive Primary Care Plus (CPC +), have shown that caring for high-risk patients can be challenging despite financial alignment that promotes coordination of care delivery [5]. Primary care teams bear most of the responsibility in caring for complex patients; in VHA over 93% of high-risk Veterans are managed by general primary care teams, despite the availability of specialized primary care teams for patients with advanced health conditions [6]. Yet, complex, high-risk patients often do not receive the most effective evidence-based care within general primary care teams [7].

Many of the evidence-based practices (EBPs) have been ineffective in the management of high-risk patients due to the lack of EBPs that properly address multimorbidity— most EBPs focus on a single problem [8, 9]. However, there are a few EBPs that have shown to be effective for high-risk patients in geriatrics and other specialized settings, such as comprehensive assessments, individualized care plans, and care coordination among the multidisciplinary team members [10–12]. Evidence also supports patient-centered approaches to support self-management for high-risk patients with competing medical and self-care demands, including shared decision making and health coaching [12–14]. However, primary care teams have not implemented these EBPs widely [15, 16]. Despite the availability of these effective practices, the most effective implementation strategies to increase uptake of EBPs for high-risk patients in primary care are unknown. EBQI has been used successfully to implement complex EBPs in VA primary care, such as the patient-centered medical home model [17] and primary care-mental health integration [18, 19]. EBQI is, in fact, a bundle of implementation strategies that emphasizes a systematic approach to developing a researcher-clinical partnership that engages national, regional, and local-level senior organizational leaders and local QI teams in adapting and implementing EBPs in the context of prior evidence and local practice conditions [17]. Core elements of EBQI include engaging multi-level multidisciplinary stakeholders in evidence-based agenda setting (developing a “QI Roadmap”), training clinical champions in QI methods to meet agenda goals, and practice facilitation [17, 18, 20, 21]. The theoretical basis underlying EBQI elements includes theories of organizational change [22–25], clinical quality improvement, [26–28] complex adaptive systems [25], and diffusion of innovation [29]; each element can be mapped to documented implementation strategies [30]. However, beyond these core elements, EBQI initiatives have widely varied in the extent and types of interactions with implementation facilitators, specifically in practice facilitation and training. Some initiatives have combined EBQI core components with individual ongoing consultation [17, 20], which can require significant researcher and quality coordinator time and resources. Other VHA EBQI initiatives have used across-site learning collaboratives (i.e., depression collaborative care [18, 19] and opioid use disorder [31]), which may require fewer resources and impact a greater number of health professionals. While both variations have been effective, individual site-level consultation has never been empirically compared with learning collaboratives; implementers lack guidance on which of these strategies are effective in what setting.

## Methods

**Fig. 1:**
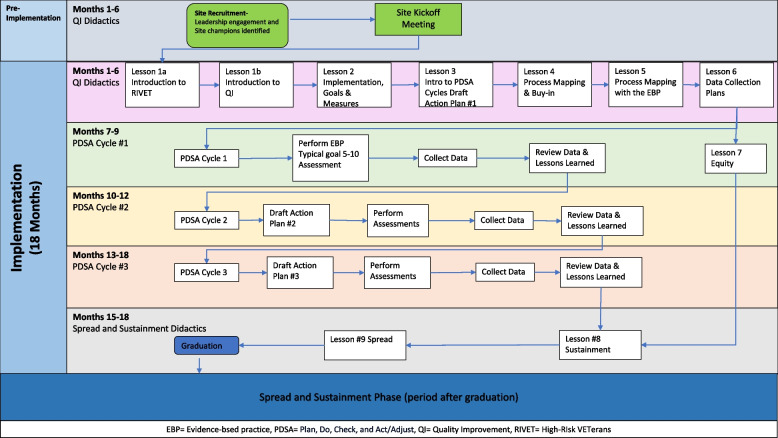
RIVET implementation timeline for each site

This study uses a mixed-methods type 3 hybrid implementation-effectiveness evaluation using a Concurrent Stepped Wedge design, evaluation of two separate interventions in different clusters, [32] to compare the two variants of EBQI aimed at increasing reach of the proposed EBPs (CACP or MAA). The Practical, Robust Implementation and Sustainability Model (PRISM) framework (Fig. 1) [33, 34] will guide the planning, implementation, and evaluation of the RIVET Program. The PRISM framework specifies contextual factors which align well with the components of our implementation strategies, and which will guide our evaluation of factors that influence Reach, Effectiveness, Adoption, Implementation, and Maintenance (RE-AIM) outcomes [34]. The Consolidated Framework for Implementation Research (CFRI) framework will identify implementation determinants, i.e., barriers and facilitators to implementation.

### Evidence‑based practices (EBPs)

We selected two EBPs that guide primary care teams to identify modifiable needs among high-risk patients through standardized assessments and utilize the expertise of the multidisciplinary staff in the primary care team: Comprehensive Assessment and Care Plan (CACP) and Medication Adherence Assessment (MAA).

#### EBP Comprehensive Assessment and Care Plan (CACP) for high-risk patients

The CACP is an assessment that helps the primary care team to develop an individualized treatment plan based on identified needs for high-risk patients of any age [35]. It was adapted from the Comprehensive Geriatric Assessment (CGA), which has been shown in multiple randomized control trials (RCTs) to lead to improved outcomes for older adults with complex care needs [36, 37]. According to meta-analyses, the CGA has consistently led to improved outcomes for frail, older adults, such as decreased mortality (OR 0.86, 95% CI 0.75–0.98), decreased readmissions (OR 0.88, 95% CI 0.79–0.98), and decreased length of stay (1.63–40.7 days in intervention group vs 1.8–42.8 days in usual care) compared to those who did not receive the CGA [38–40].

While the CGA assesses several domains that are important for complex patients, some domains may not be broadly applicable to high-risk Veterans of all ages (i.e., nutrition, vision, hearing, continence). According to our analyses, half of high-risk Veterans are younger than 65 years old and have greater psychiatric comorbidities than older high-risk Veterans [6]. We added domains to specifically assess for modifiable risk factors that are common among high-risk Veterans (e.g., transportation assistance, health literacy, behavioral health symptoms, and coordination with non-VHA healthcare systems) [41–43]. The CACP screening questions are taken from standard sources, including the National Academy of Medicine Recommendations for High-Need Patients [44] and the Protocol for Responding to and Assessing Patients’ Assets, Risks, and Experiences (PRAPARE) [45].

The CACP prompts the primary care team member to explore identified risk factors further or to refer to another team member for an in-depth assessment if needed. After the assessment, the CACP is then used to guide the development and implementation of an individualized treatment plan that addresses the patient’s health-related needs in the context of the patient’s preferences [10]. The treatment plan can be coordinated and monitored through team huddles (consisting of the primary care provider, nurses, clerk) or monthly interdisciplinary team meetings.

#### EBP Medication Adherence Assessment (MAA)

High-risk Veterans often experience complicated medication regimens, potential side effects, and other known barriers to medication adherence [46]. Medication nonadherence represents a common problem among multimorbid patients [47], and is one of the largest contributors to preventable emergency department visits and hospitalizations among high-risk Veterans. Many approaches to improving medication adherence are limited by a focus on particular diseases rather than the entire medication regimen and by a focus on the healthcare providers’ point of view, rather than on patients’ goals and agency around medication taking [48].

A standardized Medication Adherence Assessment (MAA) guides primary care teams to assess for barriers and challenges to medication adherence through open-ended questions and enables primary care teams to understand high-risk patients’ goals and preferences and better impact medication-taking behaviors across the patients’ medication conditions [12, 13]. The MAA prompts the clinician to employ specific strategies around adherence and potentially use motivational interviewing or health coaching if the patient is ambivalent about taking a medication. Health coaching has emerged as an effective, patient-centered, collaborative approach to understand patient goals and preferences and enhance patients’ adherence to modifiable health behaviors [49]. Health coaching is a type of cognitive-based behavior change technique that employs motivational interviewing and goal-setting to guide the patient to change health behaviors, such as diet, exercise, and medication adherence [49]. Meta-analyses have shown that cognitive-based behavior change techniques (e.g., health coaching) to improve medication management are associated with an effect size of 0.34 (95% CI 0.23–0.46) on improved medication adherence [50]. 

### EBQI Implementation strategies

The high-Risk VETeran (RIVET) Program will compare two variants of Evidence-Based Quality Improvement (EBQI)—practice facilitation through Individual Consultation (IC) or through Learning Collaboratives (LC) to determine which of the two is the most effective and less costly implementation strategy and explore which is better suited for which contexts.

#### Individual Consultation (IC)

Individual consultation, often described as coaching or supervision, is endorsed by implementation experts as an effective implementation strategy in and of itself. In RIVET, external facilitators from the study team will provide training to QI participants, who are front-line primary care staff, from an individual medical facility. The RIVET IC will provide regular individualized EBQI training and coaching to implement the EBP. Consultation allows experts to tailor complex skills and training to needs of the organization and to the QI participants, using active learning and providing practice opportunities [51]. It also provides QI participants with problem-solving skills and accountability [51]. Literature has demonstrated increased uptake and adherence to EBPs and increase sustainability with IC [51, 52].

#### Learning Collaboratives (LC)

Learning collaboratives are also widely used in healthcare settings and are an effective implementation strategy [30]. The RIVET learning collaboratives consists of external facilitators from the study team providing quality improvement training to QI participants from multiple sites in a group, and encourages interaction and collaboration among QI participants (e.g., providing feedback to each other). The effectiveness of learning collaboratives vary, but generally, they have demonstrated improvement in health professionals’ knowledge, problem-solving skills and attitude, and teamwork [53]. The mechanisms by which learning collaboratives may be effective include factors within an organization and factors between multiple organizations. In terms of factors within an organization, participation in learning collaboratives may increase staff confidence in using data to make decisions and to problem solve, increase accountability by making standards explicit, promote peer reflection, and facilitate teamwork, shared responsibility, and joint problem solving [53]. Mechanistic factors between organizations include normative pressure from peers, friendly competition, a platform for capacity building, and collaboration with other sites [53].

### Site selection and eligibility

We will implement the EBPs at 16 primary care sites, targeting those with high ambulatory care-sensitive hospitalization rates. Each site will be implementing a single EBP. Implementation strategies are randomized by site. Site implementation consists of four overlapping cohorts of four sites (three randomized to LC and one to IC); all will undergo 18-months of RIVET facilitation (see Figure#1). Time periods without active implementation will serve as the usual care periods for EBQI strategies. Usual care sites will receive Office of Primary Care educational campaigns and dissemination of tools for high-risk patients among primary care teams. VHA regional leaders or VHA facility leaders will select the EBP for primary care sites to implement.

### Setting

VHA primary care uses a multi-disciplinary patient-centered team-based approach (Patient-Aligned Care Teams; PACTs) where teams of health care professionals provide longitudinal care to a panel of patients [35]. Team members include a primary care provider, nurses, clerk, integrated mental health provider, social worker, and a pharmacist. Teams have access to multiple dashboards and reports for care management, including the Care Assessment Need (CAN) score [54], which describes the patient’s risk for future VA hospitalization or death by percentile.

### EBQI activities

We first engaged regional and local multidisciplinary stakeholders to discuss implementation of each EBP, such as the target patient population and the clinical staff who might perform the assessment, developing a “QI Roadmap.” QI training and practice facilitation spans 18 months of video calls with site clinical champions, selected by their facility leadership. Meetings are led by a trained RIVET external facilitator and include structured QI didactics, designing Plan-Do-Study-Act cycles, coaching, review of data, and developing structured action plans (Fig. 1). The RIVET implementation team provides quarterly data reports and regularly discuss next steps with the champions.

The same structured QI didactics are utilized for both LC and IC groups (Fig. 1). The IC sites participate in individual meetings with the clinical champions every month, on average. Learning collaboratives consist of three EBQI-LC sites that participate in monthly meetings. All sites randomly assigned to EBQI-LC participate in the same learning collaborative regardless of EBP (CACP vs MAA), as both EBPs involve the same goal, the same target population, in the same clinical setting, similar to prior initiatives [17, 31, 55].

### Data sources

Data sources include VHA Central Data Warehouse (CDW) administrative data, surveys to high-risk patients, surveys to Primary Care staff, key stakeholder interviews, implementation facilitation logs, time activity surveys, and site administrative documents.

#### EHR Administrative data

VHA CDW contains data on patient characteristics, outpatient encounters, provider types for each encounter, acute and inpatient care utilization, medication fill history, and Healthcare Effectiveness Data and Information Set (HEDIS) quality metric status. Health factor administrative data is generated by templates constructed for each EBP within the EHR. Managerial Cost Accounting (MCA) data will be utilized for the cost analyses.

#### High-risk patient surveys

Patient surveys will collect data on patient experiences among high-risk primary care patients at participating sites. Patient surveys will be mailed at the beginning and end of the 18-month active implementation phase to 500 randomly selected high-risk patients empaneled to primary care teams at each site, sampled cross-sectionally at each time period with replacement. Patient eligibility is based on the following criteria: a Care Assessment Need (CAN) score ≥ 90th percentile within the month prior to the sampling date; a visit with primary care within the last six months of the sampling date; and empanelment to the clinical champion team’s panel. When possible, patient experience and satisfaction questions were sourced from the Patient-Centered Medical Home (PCMH) version of the VHA Survey of Healthcare Experiences of Patients (SHEP), based on the Consumer Assessment of Healthcare Providers and Systems (CAHPS). Additional survey items measure direct impacts of specific EBPs (such as medication adherence), and items that may impact patient engagement in and benefit from EBPs, such as trust in their primary care provider (PCP). See Table 1 for details on included measures.

**Table 1:** RIVET QUERI patient survey measures

Measure	Source
Comprehensiveness of Care	SHEP-PCMH [56]
Care Coordination	SHEP-PCMH [56]
Self-Management Support	SHEP-PCMH [56]
Providers Discuss Medication Decisions	SHEP-PCMH [56]
Comprehensive Medication Review	One item from 3-item SHEP-PCMH Care Coordination measure [56]
Discuss Medication Adherence Strategies	Created for RIVET
Medication Adherence	Voils Medication Adherence Scale [57]
Trust in Provider	VA PACT Intensive Management (PIM) High-Risk Patient Survey [58]
Respected by Provider	VA PACT Intensive Management (PIM) High-Risk Patient Survey [58]
Satisfaction with Provider	SHEP-PCMH [56]
Satisfaction with Primary Care Clinic	Adapted from SHEP-PMCH Provider question, used in previous VA studies [59]
Patient Functional Status	NIH PROMIS Short Form Physical Function 4a measure [60]
Patient Health Literacy	Limited Health Literacy screener [61]
Social Support	From CMS Accountable Health Communities Tool [62] and Kaiser Permanente Health Assessment Questionnaire ahcm-screeningtool.pdf (cms.gov)
Social Isolation	From CMS Accountable Health Communities Tool [62] and AARP Loneliness and Connections Survey [63]
Family/Caregiver involved in medical care	Used in previous studies including with Veterans [59, 64]

*SHEP-PCMH* VA Survey of Healthcare Experiences of Patients, Patient-Centered Medical Home Version, *RIVET* High-RIsk VETerans, *PACT* Patient-Aligned Care Team, *NIH* National Institute of Health, *PROMIS* The Patient-Reported Outcomes Measurement Information System, CMS. AARP

#### Clinical staff surveys

Clinical staff surveys will be administered site-wide, to assess factors that may influence update of RIVET EBPs at the clinic, 1) other tools, resources and practices used when managing high-risk patients, and 2) exposure to RIVET EBPs (post-implementation only), and 3) confidence with practices promoted by RIVET EBPs and with overall high-risk patient care. Most items were derived from previous VA primary care staff surveys, including those conducted for the purposes of evaluating staff experiences and approaches to high-risk patient care [65]. Electronic surveys will be sent to all primary care providers, nurses, and medical assistants on eligible teams at participating sites at the beginning and end of the 18-month active implementation phase. Eligible teams include general and women’s health primary care teams, as well as any ‘specialty’ primary care teams (e.g., geriatric primary care) that the site’s champion considers eligible for EBP spread. Clinician eligibility criteria includes being a member of at least one PACT teamlet at the RIVET site; being either a physician, physician assistant, nurse practitioner, registered nurse, licensed practical or vocational nurse, a medical assistant, or a health technician; providing direct patient care at the site; and working at the primary care site for at least three months.

#### Key stakeholder interviews

Guided by the Practical, Robust Implementation and Sustainability Model (PRISM) and Consolidated Framework for Implementation Research (CFIR) frameworks, we will conduct pre- and post- semi-structured qualitative telephone interviews with key middle managers (Primary Care Medical Director, Primary Care Nursing lead, Social Work lead, Integrated Mental Health lead, Pharmacy lead) and frontline clinicians who participate in EBQI and implementation activities. The interviews will assess readiness (inner context) and its subconstructs of leadership and engagement, available resources, and access to knowledge and information; implementation climate (inner context) and its subconstructs of relative priority and values; implementation process and its subconstructs of engaging key stakeholders and executing the implementation plan; characteristics of Individuals and its subconstruct of knowledge and beliefs about the intervention; and intervention characteristics and its subconstructs of relative advantage and complexity.

#### Implementation facilitation logs

We will use templated implementation facilitation logs to collect information about participants’ attendance at EBQI activities (including facilitation sessions and other meetings), participants’ role in RIVET, plan-do-study-act cycles, ‘real time’ adaptations to the EBPs, and barriers to implementation. The Implementation facilitators and coordinator will complete the implementation facilitation logs for both EBQI-IC and EBQI-LC sites after each meeting and any contact with implementation sites and leadership.

#### Time activity surveys

We will administer weekly time surveys to the RIVET implementation team which will capture RIVET staff time spent in various implementation activities. To assess time spent on RIVET implementation activities by site participants outside of facilitation sessions and other meetings with the RIVET implementation team, we will conduct brief monthly polls via Teams during facilitation sessions.

#### Periodic reflections

Using a semi-structured interview guide, a 30-min recorded monthly meeting will be conducted with the facilitation staff to elicit information and their overall impression of implementation progress and process. The meeting will document any implementation challenges and successes, adaptations, stakeholder engagement and relevant contextual issues. We will assess contextual factors, such as organizational readiness (leadership engagement, resources, access to knowledge and information) and anticipated barriers/facilitators at each site.

#### LC and IC site administrative documents

The site administrative documents include the QI roadmap for each EBP, site action plans developed by clinical champions, meeting minutes, written reports and presentations to leadership, and attendance records.

### Measures

The Practical, Robust Implementation and Sustainability Model (PRISM) framework) [33, 34] will guide the planning, implementation, and evaluation of the RIVET Program. The PRISM framework specifies contextual factors which align well with the components of our implementation strategies, and which will guide our evaluation of factors that influence Reach, Effectiveness, Adoption, Implementation, and Maintenance (RE-AIM) outcomes [34]. We will use mixed methods to evaluate RE-AIM outcomes, EBP fidelity, implementation strategy fidelity, adaptations, costs, benefits, and value.

#### Reach

Reach is defined in this study as the proportion of high-risk patients on each study team’s panel that received one of the EBPs during the 18-month implementation period. See Table 2 for details on included reach measures. We define “high-risk patients” using a VHA-specific risk score called the Care Assessment Need score, previously developed and validated by VA through machine-learning to predict a patient’s risk for a VHA hospitalization or mortality [54]. We define ‘receipt’ as having the EBP assessment at least partially documented in the electronic health record (EHR). We will also examine the patient characteristics (sociodemographic, Elixhauser comorbidity score [66]) of eligible patients who did vs. did not receive the EBP.

**Table 2:** Reach and effectiveness outcomes metrics

	**Reach** **(Primary Outcome)**	**Proximal Care Process Impacts**	**Patient and Provider Impacts**	**Clinical Quality and Outcomes**
EBP 1 CACP	Proportion of site-assigned patients in the top 10% of CAN-hospitalization scores that receive the EBP	• Number and type of referrals generated • Referrals completed by patient • Number of primary care social worker, pharmacist, nurse, integrated MH encounters	• Comprehensiveness of Care (Patient Survey^a^/SHEP) • Self-Management Support (Patient Survey^a^/SHEP) • Primary Care provider experience and perceived support for high-risk patient care (Clinician Survey)	• VHA ACS-related and total acute hospitalizations • VHA ACS-related and total emergency department visits
EBP 2 MAA		• Number and type of referrals or actions documented receiving the EBP • Number of primary care pharmacist, social worker, nurse, integrated MH encounters	• Providers Discuss Medication Decisions/Medication Comprehensiveness (Patient Survey^a^/SHEP) • Self-Management Support (Patient Survey^a^/SHEP)	• Medication adherence (Patient Survey^a^) • Adherence for DM, HTN, HL, MH medications (VHA Pharmacy Data) • HEDIS measures for DM, HTN, HL management

*ACS* Ambulatory care-sensitive, *EBP* Evidence-based practice, *CACP* Comprehensive Assessment and Care Planning, *CAN* Care Assessment Need, *DM* diabetes mellitus, *HTN* hypertension, *HEDIS* Healthcare Effectiveness Data and Information Set, *HL* hyperlipidemia, *MAA* Medication Adherence Assessment, *MH* Mental Health, *PDC* proportion of days covered, *SHEP* Survey of Healthcare Experiences of Patients

^a^Refers to patient survey referenced in the Methods

#### Effectiveness

EBP effectiveness measures will include type of referrals or actions generated by each EBP, and whether they were completed, as measured by EHR template use, administrative consult data, and encounters. We will also use administrative EHR data to measure number of patient encounters with primary care team members (social work, pharmacist, nurse, integrated mental health), as an indicator of engagement of primary care teams for high-risk patient care. Impacts of RIVET on patient experience will be evaluated with a patient survey that includes measures (described above) of satisfaction and access to resources and support for caring for high-risk patients. We will also assess clinical performance metrics that are expected to be directly impacted by each EBP. For both EBPs, we will measure acute care utilization, such as emergency department (ED) visits, total acute and ambulatory care-sensitive (ACS)-related hospitalizations. For MAA, we will also measure adherence to medications for common chronic conditions, such as hypertension and diabetes. See Table 2 for details on included effectiveness measures.

#### Adoption

We will assess adoption by measuring number and proportion of staff trained on each EBP, and how many and which types of staff delivered each EBP (representativeness), using administrative training records and administrative clinical data for each EBP.

#### Implementation fidelity

We will assess implementation strategy fidelity using the EBQI fidelity assessment tool [55]. The fidelity assessment tool draws from data collected from key stakeholder interviews, implementation facilitation logs, administrative documents, and weekly time diaries (described below). We will apply criteria to rate sites as high, medium, or low fidelity on the EBQI elements. Using the implementation facilitation logs, we will assess participation in the EBQI activities by frontline providers, staff, and leadership.

#### Maintenance

We assess maintenance by the extent to which EBPs are implemented after practice facilitation ends (e.g., about 18 months). We also plan to study if the EBPs are spread to other primary care teams within the site and to other sites within the healthcare system.

## Outcomes and data analyses

To analyze our primary outcome, receipt of each EBP among top 10th percentile high-risk patients during the 18-month implementation period (Reach), we will model uptake of both practices in our Concurrent Stepped Wedge Design as a multilevel hierarchical model with a repeated cross-sectional data structure in which sites are followed over time. In this design, the data structure includes patients at level 1 (where Reach, the main outcome of interest, in measured), nested within time at level 2, and nested within sites at level 3. The main predictors will be site implementation strategy assignment (EBQI-IC vs. EBQI-LC vs Usual Care) over time (measure as the number of quarter). We will first describe differences in trends using unadjusted analyses, then add covariates for patient characteristics (i.e., age, sex, Elixhauser comorbidity score [66]) and site-level covariates (i.e., number of unique patients served in primary care, rural vs urban). Secondary analyses of Reach will include models by key patient subgroups, including those hospitalized in the 90 days prior to the quarter examined, and models with an interaction term for assigned implementation strategy by EBQI fidelity level (as defined above), to examine how implementation strategy fidelity may have impacted Reach.

To analyze our secondary outcomes of care processes, patient experiences, provider experiences, and performance measures (Effectiveness), we will first model unadjusted trends in outcomes over time for each EBP, by site implementation strategy assignment. We will then model outcomes using two or three-level (depending on whether the metric is measured at the patient or site level) hierarchical models based on the concurrent Stepped Wedge Design using repeated cross-sectional data, adjusted for the same covariates included in the models for our primary outcome. In models of medication adherence, we will add covariates for medication regimen complexity, as indicated by total number of classes of medication prescribed for the condition of interest, and patient co-pay status. If we find differences in outcomes, we will perform mediation analyses that consider how Reach and EBP fidelity may have mediated outcomes [67].

To assess differences in adoption between EBQI-IC and EBQI-LC sites, we will use bivariate analyses to compare the number and proportion of staff trained on each EBP, and how many and which types of staff delivered each EBP. To assess differences in implementation strategy fidelity among sites, we will compare the number of sites with high, medium, and low fidelity on each element of EBQI, as well as overall fidelity to EBQI. Similarly, we will compare number of sites with overall high, medium, or low EBP fidelity among EBQI-IC vs EBQI-LC sites.

We will qualitatively assess the impact of contextual factors on implementation, using a matrix analysis approach [68] to explore a priori themes based on the interview guides, but also allow for emergent themes. Specifically, we will code and analyze interview data for core elements of the EBQI implementation strategy and for contextual features, intervention characteristics, and implementation infrastructure guided by the PRISM framework [33]. Two trained qualitative analysts will construct and validate the codebook [69]. Using this codebook, one analyst will code all interviews, and a second qualitative analyst will review all coding. After generating a report for all codes, they will use a matrix analysis approach [68] to populate a participant-by-theme matrix and create site level summaries for each theme to facilitate comparisons between EBQI-IC and EBQI-LC sites. To ensure rigor, summaries will also be reviewed, compared with the original data, and discussed by at least two analysts to reach consensus for any discrepancies. Finally, we will link this qualitative matrix with site-level implementation strategy and EBP fidelity measures to compare how specific contextual features, intervention characteristics, and implementation infrastructure impacted fidelity.

### Return-On-Investment (ROI) Analyses

We will conduct a budget impact analysis to inform the sustainability of each EBP. Following the VHA recommendation to evaluate cost of projects [70], we will identify the relevant costs associated with implementation of the EBP, the EBP itself, and the consequences of the EBP (e.g., healthcare utilization). Using a micro-costing approach [71], we will collect costs for EBQI-IC and EBQI-LC sites, measuring: 1) implementation costs as one-time costs to develop the intervention; and 2) intervention and downstream costs as costs that would be incurred by facilities adopting the EBPs (e.g., site participants; RIVET implementation team members’ time spent in training, meetings and preparing for meetings; staff time performing the intervention). To capture RIVET implementation team staff time spent in various implementation activities, data will be collected through the implementation facilitation log, administrative documents, and weekly time surveys. For both EBPs, clinical staff will document the estimated time spent to complete EBP through an EHR template. Finally, we will identify the costs of healthcare utilization which may be impacted by the implementation of these EBPs from the VA administrative data, such as change in outpatient utilization (e.g., primary care, social work, mental health, pharmacy) and inpatient utilization. This will be done by estimating the excess cost for patients exposed to the EBP compared to a control group of unexposed patients using multivariable regression models to control for measured confounders. We will use generalized linear models for continuous data and two-part models for semi-continuous data with distributional families chosen to best fit the data. If preliminary data reveals that medication adherence changed with MAA implementation, we will also include changes in pharmacy costs.

We will measure whether EBPs were maintained during each sites’ sustainment period (time period following the 18-month active implementation period), how EBPs spread within the original sites and to new sites, and what factors are associated with maintenance and spread. We will also assess adaptations made to the EBPs in response to changing VHA context.

For sustainment and spread, we will continue to assess receipt of each EBP among top 10% CAN score patients (Reach) longitudinally during the sustainment period by measuring 6 and 12 months after the active implementation phase. We will incorporate Adoption measures to assess spread within the implementation site and in new sites by measuring how many additional staff were trained on each EBP, proportion of staff trained on each EBP, and which types of healthcare staff delivered each EBP (representativeness). We will compare EBQI-IC and EBQI-LC sites to determine which implementation strategy is most effective for sustaining the EBPs. Using the matrix analysis approach described above, we will analyze qualitative interview data to explore the role of contextual factors on sustainment, and the “fit” between context, intervention, and implementation strategy on sustainability.

## Discussion

The RIVET project aims to implement two evidence-based assessments to improve the management of the high-risk patient population within VHA primary care using two EBQI strategies. This work will add to a much needed body of literature evaluating the effectiveness of different approaches to EBQI to implement EBPs within primary care [1]. It is the first study to compare the effectiveness of EBQI conducted with individual sites (EBQI-IC) vs conducted with groups of sites as a learning collaborative (EBQI-LC) and to compare which implementation strategy is most effective under various contexts accounting for unique barriers, facilitators, and adaptations. Additionally, RIVET will provide evidence on which of the two strategies is the most cost-efficient strategy. Comparing EBQI-LC and EBQI-IC will allow our VHA primary care leaders to tailor the implementation strategy to the primary care context in preparation for widespread implementation. While our project focuses on EBPs for high-risk patients, we anticipate that this comparison of EBQI strategies can inform those implementing EBPs with other primary care patient populations.

The EBPs aim to systematically identify modifiable risk factors within primary care for patients with complex needs—enabling primary care to provide comprehensive and holistic care. Furthermore, by selecting the most effective, less burdensome, and less costly implementation strategy ensures greater buy-in from clinical leadership interested in offering advanced primary care and frontline staff who are often overwhelmed with clinical demands and chronic staffing shortages.

We anticipate several potential challenges to optimal implementation. The major challenge for the RIVET project is that, as with any project embedded in pragmatic healthcare system operations, it is vulnerable to national and local VHA contextual factors. Specifically, success of the project can be compromised by VHA staffing changes within the sites and study teams. In addition, since primary care teams are tasked with a wide variety of care, new health system initiatives and external circumstances (e.g., pandemic-induced changes in care delivery) can unexpectedly compete with high-risk patient care priorities. In addition, the active implementation period requires 18-month engagement from clinical champions to learn EBQI practices and to properly use the EBP in their routine care of high-risk patients. Finally, RIVET EBQI IC and LC sessions will be held virtually. Although most provider training has moved to virtual modalities post-Covid, the best methods to keep staff engaged may vary over time and setting. The RIVET project will not only implement EBP tools that will help better manage high-risk Veterans at 16 VA sites but will provide the tools and evidence on the best implementation strategies for primary care staff at VHA working to improve high-risk patient care and primary care delivery.

## Data Availability

Data sharing is not applicable to this article as no datasets were generated or analyzed during the current study.

## Abbreviations

ACS: Ambulatory care-sensitive
CACP: Comprehensive Assessment and Care Planning
CAHPS: Consumer Assessment of Healthcare Providers and Systems
CAN: Care Assessment Need
CFIR: Consolidated Framework for Implementation Research
CGA: Comprehensive Geriatric Assessment
CPC +: Comprehensive Primary Care Plus
CDW: Central Data Warehouse
EBP: Evidence-based practice
EBQI: Evidence-Based Quality Improvement
ED: Emergency department
EHR: Electronic health record
HEDIS: Healthcare Effectiveness Data and Information Set
IC: Individual coaching
LC: Learning collaborative
MAA: Medication Adherence Assessment
MCA: Managerial Cost Accounting
OPC: Office of Primary Care
PACT: Patient-Aligned Care Team
PCMH: Patient-Centered Medical Home
PCP: Primary care provider
PRAPARE: Protocol for Responding to and Assessing Patients’ Assets, Risks, and Experiences
PRISM: Practical, Robust Implementation and Sustainability Model
QUERI: Quality Enhancement Research Initiative
RCT: Randomized control trial
RE-AIM: Reach, Effectiveness, Adoption, Implementation, and Maintenance
RIVET: High-RIsk VETerans
ROI: Return-On-Investment
SHEP: Survey of Healthcare Experiences of Patients
VHA: Veterans’ Health Administration
