# Correction: Implementing evidence-based practices to improve primary care for high-risk patients: study protocol for the VA high-RIsk VETerans (RIVET) type III effectiveness-implementation trial

**DOI:** 10.1186/s43058-024-00623-7

**Published:** 2024-08-01

**Authors:** Elvira E. Jimenez, Ann‑Marie Rosland, Susan E. Stockdale, Ashok Reddy, Michelle S. Wong, Natasha Torrence, Alexis Huynh, Evelyn T. Chang

**Affiliations:** 1 Center for the Study of Healthcare Innovation, Implementation & Policy (CSHIIP), VA Greater Los Angeles Healthcare System, 11301 Wilshire Blvd, Los Angeles, CA 90073 USA; 2 Department of Neurology, David Gefen School of Medicine, University of California Los Angeles (UCLA), 760 Westwood Plaza, Los Angeles, CA 90095 USA; 3 Center for Health Equity Research and Promotion (CHERP), VA Pittsburgh Healthcare System, 1 University Dr, Pittsburgh, PA 15240 USA; 4 Caring for Complex Chronic Conditions Research Center &, Division of General Internal Medicine, School of Medicine, University of Pittsburgh, 3550 Terrace St, Pittsburgh, PA 15213 USA; 5 Department of Medicine, Division of General Internal Medicine, Harborview Medical Center, University of Washington, 325 Ninth Ave, Box 359780, Seattle, WA 98104 USA; 6 Center for Veteran-Centered and Value-Driven Care, Puget Sound Health Care System, 1660 S Columbian Way, Seattle, 98108 USA; 7 Division of General Internal Medicine, Department of Medicine, David Gefen School of Medicine, UCLA, 740 Charles E Young Dr S, Los Angeles, CA 90095 USA; 8 Department of Psychiatry and Biobehavioral Sciences, David Geffen School of Medicine, University of California Los Angeles (UCLA), 760 Westwood Plaza, Los Angeles, CA 90095 USA

**Correction: Implement Sci Commun 5, 75 (2024)**

**https://doi.org/10.1186/s43058-024-00613-9**

Following the publication of the Original Article [1], the authors reported wrong affiliations concerning the 1st and 3rd authors. Affiliation 8 Department of Psychiatry and Biobehavioral Sciences, David Geffen School of Medicine, University of California Los Angeles (UCLA), 760 Westwood Plaza, Los Angeles, CA, 90095, USA was also missing in the Original Article.

**Incorrect**

Elvira Jimenez is affiliated with Affiliations 1, 2, and 4.

Susan E. Stockdale is affiliated with Affiliations 1, 2.

**Correct**

Elvira Jimenez is affiliated with Affiliations 1 and 2.

Susan E. Stockdale is affiliated with Affiliations 1 and 8.

The author group has been updated above and the Original Article has been corrected.
